# Niche-Specific Requirement for Hyphal Wall protein 1 in Virulence of *Candida albicans*

**DOI:** 10.1371/journal.pone.0080842

**Published:** 2013-11-08

**Authors:** Janet F. Staab, Kausik Datta, Peter Rhee

**Affiliations:** Johns Hopkins School of Medicine, Johns Hopkins University, Baltimore, Maryland, United States of America; University of Wisconsin Medical School, United States of America

## Abstract

Specialized *Candida albicans* cell surface proteins called adhesins mediate binding of the fungus to host cells. The mammalian transglutaminase (TG) substrate and adhesin, Hyphal wall protein 1 (Hwp1), is expressed on the hyphal form of *C. albicans* where it mediates fungal adhesion to epithelial cells. Hwp1 is also required for biofilm formation and mating thus the protein functions in both fungal-host and self-interactions. Hwp1 is required for full virulence of *C. albicans* in murine models of disseminated candidiasis and of esophageal candidiasis. Previous studies correlated TG activity on the surface of oral epithelial cells, produced by epithelial TG (TG1), with tight binding of *C. albicans* via Hwp1 to the host cell surfaces. However, the contribution of other Tgs, specifically tissue TG (TG2), to disseminated candidiasis mediated by Hwp1 was not known. A newly created *hwp1* null strain in the wild type SC5314 background was as virulent as the parental strain in C57BL/6 mice, and virulence was retained in C57BL/6 mice deleted for Tgm2 (TG2). Further, the *hwp1* null strains displayed modestly reduced virulence in BALB/c mice as did strain DD27-U1, an independently created *hwp1*Δ/Δ in CAI4 corrected for its *ura3*Δ defect at the *URA3* locus. Hwp1 was still needed to produce wild type biofilms, and persist on murine tongues in an oral model of oropharyngeal candidiasis consistent with previous studies by us and others. Finally, lack of Hwp1 affected the translocation of *C. albicans* from the mouse intestine into the bloodstream of mice. Together, Hwp1 appears to have a minor role in disseminated candidiasis, independent of tissue TG, but a key function in host- and self-association to the surface of oral mucosa.

## Introduction

*Candida albicans* is often found in the oral cavity and gastrointestinal (GI) tract of healthy individuals as a member of the normal flora. Impairment of the immune system allows for the fungus to overgrow, germinate and produce filamentous cells capable of invading the local mucosal surface. Germination and production of filamentous hyphal cells is accompanied by the expression of adhesion proteins that facilitate host cell interactions. Hyphal wall protein 1 (Hwp1; Accession number AAC96368) [1], Als1p (Agglutinin-like sequence 1 protein) and Als3p are involved in the binding of *C. albicans* to oral epithelial cells [2–6]. 

Hwp1 participates in the formation of covalent bonds to primary amines and to a buccal epithelial cell (BEC) surface protein catalyzed by human transglutaminases (TGs) [1,7,8]. TGs are multi-functional enzymes widely distributed in the body that modify glutamyl residues in proteins [9–11]. These enzymes catalyze the formation of isodipeptide bonds between an acceptor glutamine residue and a donor lysine residue in other proteins, a reaction known as transamidation. TG1 (keratinocyte transglutaminase) is active on the surface of BECs [12] where it functions in assembly of the cornified cell envelope, a scaffold of cross-linked proteins that gives mucosal cells their barrier properties. We found the participation of Hwp1 in the formation of a stabilized (heat and detergent-resistant) TG-dependent adhesion to BECs [1], and speculated that TG1 on the surface of BECs catalyzed this reaction. In support of this conclusion, an *hwp1*Δ/Δ strain is unable to cause oropharyngeal candidiasis in mice [13]. However, we were unable to verify the role of TG1 directly as Tgm1^-/-^ mice die of dehydration soon after birth [14]. 

Although a mechanism of adhesion based on transglutaminase activity on BECs could explain the requirement for Hwp1 in oral candidiasis, the functional requirement for Hwp1 in disseminated candidiasis in mice [1,15] where active TG1 is not expressed [16] is not clear. TG2 (tissue transglutaminase) is widely distributed in the body (e.g. liver, kidneys, extracellular matrix, muscle) [9,10,16] therefore, we hypothesized that TG2 may have a role in virulence in a disseminated candidiasis animal model. *In vitro*, both native and recombinant Hwp1 are substrates for guinea pig liver TG2 [1], therefore, it is plausible that tissue TG2 catalyzes transamidation reactions involving Hwp1. To test this hypothesis, we created a new *hwp1* null strain (*hwp1*Δ/Δ) in the wild type *C. albicans* strain SC5314 using the *flp* recombinase method [17] to avoid *URA3*-associated confounding effects [18–20]. We tested the virulence of the new *hwp1*Δ/Δ strain in wild type C57BL/6 and in Tgm2^-/-^ mice, and did not observe a TG2-dependent requirement for Hwp1. We re-tested the virulence of the newly created null strain in BALB/c mice as before [15] and observed that virulence was attenuated, and reintroducing *HWP1* at its native locus did not restore full virulence of the reconstituted strain (*hwp1*Δ/+*HWP1*). In light of these results, other Hwp1-phenotypes were tested with the newly created deletion strain. Biofilm formation was deficient in the *hwp1*Δ/Δ strain as reported by others [21,22] and restored upon reintroducing a functional copy of *HWP1* at its native locus. The knock-out strain was also deficient in causing disease in an oropharyngeal model of candidiasis [23]. Lastly, we examined the role of Hwp1 in gut translocation of *C. albicans* to the bloodstream of C57BL/6 mice using the experimental model previously developed [24]. This infection model mimics a common infection route in at-risk candidiasis patients [24–26], and the role of Hwp1 in this model in unknown. The *hwp1*Δ/Δ strain was capable of colonizing the mouse gut to equal levels as *HWP1*+ strains. However, the *hwp1* null strain was less virulent relative to the wild type or the reconstituted strain in this model, thus defining a new phenotype for Hwp1 in murine candidiasis.

Our results suggested that Hwp1 has adapted *C. albicans* for localized infections of the oral mucosa and is less important for disseminated disease. The fungus is still capable of invading kidneys and livers during invasive disease in the absence of Hwp1 or TG2. The wild type genetic background of SC5314 has allowed us to discern subtle *in vivo* phenotypes that were previously masked, perhaps due to unknown *in vivo* fitness defects of the previous parental strain (CAI4 [27]). Our results suggest that Hwp1 engenders *C. albicans* with niche-specific capabilities to inhabit the oral cavity where fungal-host and fungal-fungal interactions are needed for successful colonization and localized superficial tissue invasion that typifies oropharyngeal candidiasis. 

## Materials and Methods

### Ethics statement

All animal studies were approved by the Johns Hopkins University Animal Care and Use Committee (ACUC). The approved protocol was in compliance with the Animal Welfare Act regulations and Public Health Service (PHS) Policy. For virulence/survival studies, *C. albicans*-infected mice were closely monitored for signs of distress. Moribund mice exhibiting signs of illness (ruffled fur, lethargy) were humanely sacrificed by CO_2_ inhalation according to the recommendations of the Johns Hopkins University ACUC. The studies were performed under the ACUC protocol number MO11M261.

### *Candida albicans* strains

Clinical isolate, SC5314 [28], was used as source for genomic DNA and for the construction of *HWP1* deletion mutants. The *hwp1*Δ/*hwp1*Δ strain constructed in CAI4 [27], DD27-U1[29], was kindly provided by William Fonzi (Georgetown University, Washington, DC). The organisms were stored at -80^O^C and cultured in liquid or agar-containing plates of yeast peptone dextrose (YPD) medium at 30°C according to standard methods [30].

### *Candida albicans* deletion strains

**Figure 1:**
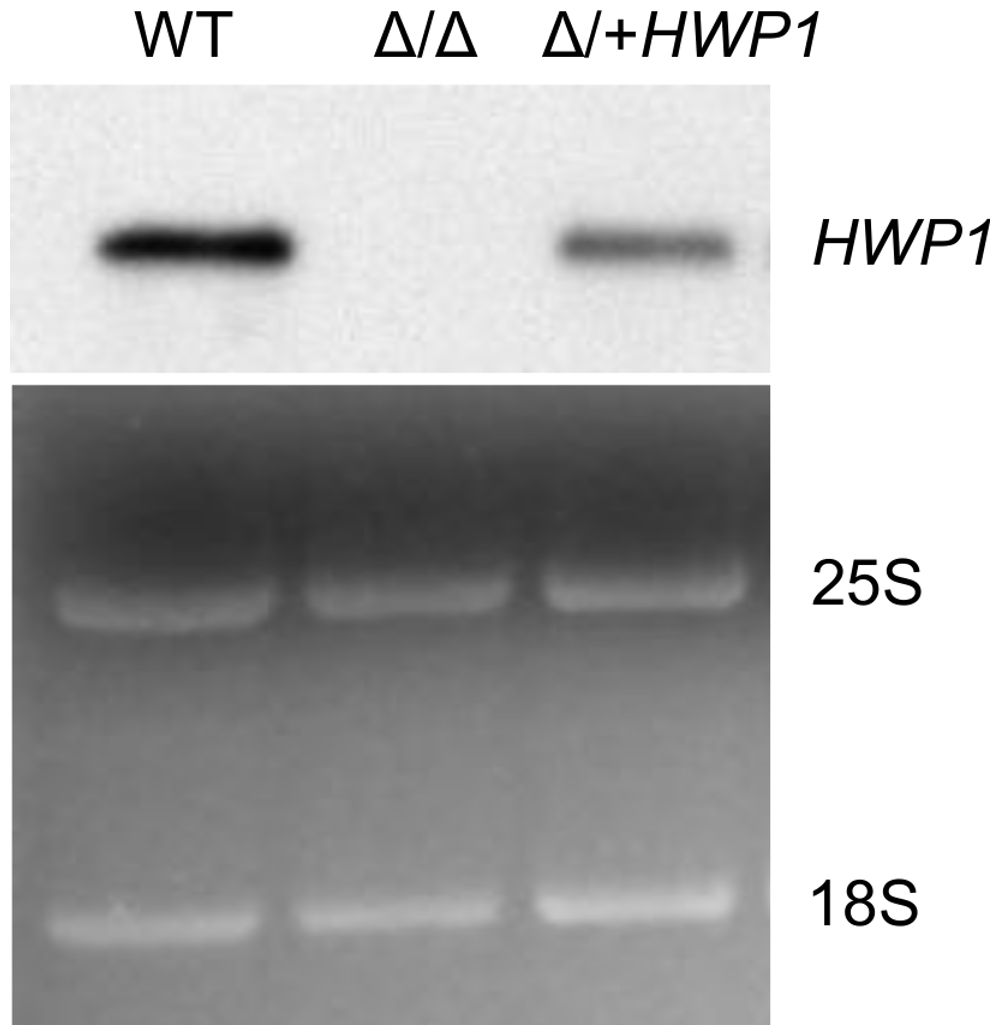
Deletion and reconstitution of *HWP1* expression in SC5314. Northern blot analysis of the parental SC5314 (WT), *hwp1* null SCH1211 (Δ/Δ) and *HWP1* put-back HR615 (Δ/+*HWP1*) strains. Five µg of total RNA was separated in a standard formaldehyde gel and transferred to a nylon membrane prior to hybridization with a biotinylated riboprobe to 5’ *HWP1* sequences (top panel). Lower panel shows the rRNA bands stained with ethidium bromide as loading control.

SC5314 was used as parent to delete the coding region for *HWP1* using the *SAT1* flipper system for gene disruption in *C. albicans* [17,31]. Plasmid pSFS1 (provided by Joachim Morschhäuser, University of Würzburg, Würzburg, Germany), a derivative of pSFS2 [17], was used to construct the disruption cassette for *HWP1* (orf19.1321; www.candidagenome.org) as follows: the region between bp -491 to +35 (relative to ATG; 5’ amplicon) was amplified using the PCR from SC5314 genomic DNA with oligonucleotides H1Ap and H2Xh (Table S1), and the region between bp +1860 to +2249 (3’ amplicon) of *HWP1* was amplified with oligonucleotides H3SII and H4 using *Platinum Taq* High Fidelity (Invitrogen/Life Technologies, Grand Island, NY) according to the manufacturer’s recommendations. The 3’ amplicon contained sequences downstream of the polyadenylation site of the *HWP1* mRNA [32], across a SacI site. The *HWP1* rescue fragment was also generated by the PCR (bp -362 to bp 517 downstream of the stop codon) using oligonucleotides H1Ap and H5Xh to add an ApaI site and an XhoI site at the 5’ and 3’ ends of the amplicon, respectively. The disruption plasmid was constructed by first cloning the 3’ *HWP1* amplicon between the SacII and SacI sites of pSFS2 to generate pSFSH3. This plasmid was then used to clone both the 5’ *HWP1* amplicon between the ApaI and XhoI sites, and the rescue fragment between the ApaI and SalI sites. The cloned sequences were verified by sequencing for authenticity and PCR-induced errors. The disruption plasmid, pSFSH35, contained 5’ and 3’ *HWP1* sequences interrupted by the *SAT1* flipper cassette, while the rescue plasmid, pSFSH329, contained *HWP1* sequences up- and downstream of the coding region followed by the *SAT1* flipper cassette and 3’ *HWP1* sequences. Gel-purified linear disruption fragment generated by digesting pSFSH35 with KpnI and SacI was used to transform SC5314 by electroporation [17]. Transformants were selected on YPD plates containing 250 µg/mL of nourseothricin (clonNAT, Werner BioAgents, Jena, Germany) at 30°C for 2 days. Two independent transformants integrated the disruption cassette at a single allele of *HWP1*, and were genotypically *HWP1*/*hwp1*Δ::*SAT1*-*FLP* determined by Southern blot analysis (see Figure S2). Both transformants were cultured in YPD for 24 h at 30°C in the presence of 0.4% BSA (Sigma-Aldrich, St. Louis, MO; product number B4287) to induce loss of the *SAT1* cassette via intramolecular recombination at the *FRT* sites by the site-specific recombinase FLP, and to re-establish nourseothricin sensitivity. Loss of the *SAT1*-*FLP* cassette was verified by Southern blotting, and both single disruption derivatives (*HWP1*/*hwp1*Δ::*FRT*) were subjected to a second round of transformation with the linearized deletion construct. Integration of the disruption cassette at the intact *HWP1* allele was verified by Southern blotting, and two derivatives were grown in YPD 0.4% BSA to induce loss of the *SAT1-FLP* cassette at the second *HWP1* allele. Nourseothricin sensitive strains were subsequently verified by Southern blotting. The double disruption strain SCH1211 (*hwp1*Δ::*FRT*/*hwp1*Δ::*FRT;* “*hwp1*Δ/Δ”) was used as recipients for the rescue DNA fragment harbored in pSFSH329 (digested with BssHII release the rescue *HWP1* gene fragment). Integration of the rescue DNA fragment at a single (*hwp1*Δ::*FRT* allele was verified by Southern blotting. Reintegrant derivatives were grown in YPD 0.4% BSA to generate strains sensitive to nourseothricin and with a single functionally corrected *HWP1* verified by northern blotting or reverse transcription-PCR (RT-PCR) (Figure 1 and data not shown).

### Southern blot analysis

Genomic DNA was prepared from overnight cultures using a MasterPure Yeast DNA Purification Kit (Epicentre Biotechnologies, Madison, WI). Three to five µg of DNA was digested to completion with NdeI (Invitrogen/Life Technologies, Grand Island, NY) and the fragments separated in an agarose gel as per standard methods. The DNA fragments were blotted onto a BrightStar-Plus (Ambion/Life Technologies) nylon membrane and probed with a biotinylated (BrightStar Psoralen-Biotin labeling kit, Ambion/Life Technologies) *HWP1* amplicon (nt -491 to +35 relative to the ATG) overnight at 42°C in ULTRAHyb solution (Ambion/Life Technologies). DNA bands were visualized by incubating the membrane with Strepavidin conjugated to alkaline phosphatase and chemiluminescence reagents (BrightStar BioDetect Kit, Ambion/Life Technologies), followed by exposure of the membrane to X-ray film (Kodak BioMax XAR).

### Northern blotting

Total RNA was prepared using the hot acid phenol method [33] from *C. albicans* germ tubes grown in YPD with 10% bovine serum for two hr at 37°C. Five µg of total RNA was separated in a standard formaldehyde gel and transferred to a charged nylon membrane (BrightStar-Plus, Amibon/Life Technologies). The membrane was probed with a biotinylated (BrightStar Psoralen-Biotin Kit, Ambion/Life Technologies) antisense transcript (riboprobe) containing *HWP1* sequences from +11 to +623 relative to the ATG start site generated according to the manufacturer’s recommendations (MAXIscript Kit, Ambion/Life Technologies). The membrane was hybridized overnight at 68°C in ULTRAHyb solution (Ambion/Life Technologies). RNA bands were visualized by incubating the membrane with Strepavidin conjugated to alkaline phosphatase and chemiluminescence reagents (BrightStar BioDetect Kit, Ambion/Life Technologies), followed by exposure of the membrane to X-ray film (Kodak BioMax XAR).

### Germ tube TG assays

Expression of Hwp1 on the surface of germ tubes was visualized by cross-linking 5-(biotinamido)pentylamine (5-BPA) (Pierce/Thermo Scientific, Rockford, IL) as before [1] with slight modifications. Human recombinant TG2 (hTG2; Zedira, Darmstadt, Germany) (12.5 U/mL) was used in 400 µL reactions containing 2 x 10^7^ germ tubes in reaction buffer (100 mM Tris-HCl pH 7.5, 10 mM CaCl_2_, 1 mM DTT and 1 mM EDTA, 25 µM 5-BPA) for 15 min at 37°C. The reactions were stopped by adding 1.0 mL of 10 mM EDTA, pH 8.0, and the germ tubes washed twice with dH_2_O. Germ tubes (1 x 10^5^) were spotted onto poly-L-lysine coated slides, allowed to air dry and incubated with Streptavidin conjugated to FITC (Invitrogen/Life Technologies) at 1:50 dilution in phosphate buffered saline (PBS, GIBCO/Life Technologies). After 30 min incubation at 37°C, the slides were washed in PBS and rinsed with water. A drop of VECTASHIELD mounting medium with DAPI (VECTOR Laboratories Inc, Burlingame, CA) was added to the germ tubes before observing under epifluorescence using the FITC or DAPI excitation cubes (Zeiss LSM 410 confocal microscope; Carl Zeiss Microscopy, Jena, Germany). Cell images were acquired with an Olympus camera running cellSense 1.6 software (Olympus America). Captured images were minimally manipulated with ImageJ64 (v.1.46r; open-source software; http://imagej.nih.gov.ij).

### Virulence studies

Female BALB/c mice (6-8 weeks) were purchased from Charles River Laboratories (Frederick, MD) one week prior to experiments to allow the mice to acclimatize to the housing at JHU. C57BL/6 Tgm2^-/-^ [34] breeder mice were purchased from the European Mouse Mutant Archive (Munich, Germany) and bred in-house to generate animals for our studies. Wild type female (6-8 weeks) C57BL/6 controls were purchased from The Jackson Laboratory (Bar Harbor, ME) at least one week prior to experiments. 

Disseminated candidiasis studies [35] were performed as follows: *C. albicans* strains were grown in YPD with vigorous shaking for approximately 24 h at 30°C, washed twice with PBS and adjusted to an OD_600_ of 0.3-0.32 (3.5-4.0 x 10^6^ cfu/mL) in sterile PBS. We found that adjusting the yeast suspensions to OD_600_ of 0.3-0.32 (vs. counting by hemocytometer) produced consistent results across experiments. One hundred microliters of the yeast suspensions was injected into the tail veins of mice, and these observed for signs of illness over a 10-14 day period. Moribund mice were humanely euthanized by CO_2_ inhalation. For histological observations, C57BL/6 and Tgm2^-/-^ mice injected with SCH1211 (*hwp1*Δ::*FRT*/*hwp1*Δ::*FRT*) were sacrificed at day 3 post inoculation. Kidneys were fixed in buffered formalin, embedded in Paraffin, and sections stained with Periodic-acid Schiff (PAS) to observe the fungal elements at the site of infection. 

The role of Hwp1 in gut-translocation and dissemination of *C. albicans* was tested in mice as previously described [24] (University of Texas Southwestern Medical Center, Dallas, TX) with one modification. Briefly, 6-8 week old female C57BL/6 mice were gut-colonized with *C. albicans* after first reducing the GI bacterial and fungal flora with penicillinG (1500 U/mL) and streptomycin (2 mg/mL), and fluconazole (250 µg/mL) in their drinking water. After verifying the depletion of the gut normal flora, the drinking water was replaced with water containing a suspension of *C. albicans* at 10^7^ cfu/mL plus penicillinG and streptomycin as above for 5 days. After verifying gut-colonization of *C. albicans* as CFU/g of stools, the mice were immunosuppressed by four (vs. three in the original protocol) intraperitoneal injections of cyclophosphamide (150 mg/kg/dose) every other day. Groups of mice (n=7) were observed for signs of illness prior to sacrifice to determine the fungal burden in the livers. Serial dilutions of homogenized livers were spread onto YPD agar with vancomycin at 10 µg/mL and gentamycin at 100 µg/mL, trypicase soy agar (TSA) and MacConkey’s agar plates (microbiological media from Becton-Dickinson). Creamy white colonies were enumerated to determine CFU/g of liver.

### *Candida* biofilms

*C. albicans* biofilms were produced on silicone squares as before [22] with slight modifications. Silicone squares (1.5 x 1.5 cm) (Bentec Medical, Inc., Woodland, CA) were conditioned overnight at 37°C in bovine calf serum (Hyclone), rinsed with PBS and placed in non-tissue culture 6-well plates containing 2 mL of Spider medium (1% nutrient broth; Remel/Thermo Scientific; 1% mannitol and 2% potassium monophosphate, the latter two reagents from Sigma-Aldrich). *C. albicans* cultures were grown for approximately 18 h in YPD at 30°C, washed with PBS and adjusted to an OD_600_ of 1.0 in Spider medium. Two mL of each yeast culture was added to the silicone squares to produce a final cell suspension at OD600 equal to 0.5. After 90 min incubation at 37°C with shaking at 60 rpm, the silicone squares were briefly rinsed in PBS and transferred to new 6-well plates containing 4 mL of fresh Spider medium. The plates were further incubated at 37°C for 48 h with shaking. The silicone squares were subsequently rinsed in PBS and set to dry for 2-3 days at room temperature inside a fume hood prior to weighing. The biofilms were generated twice in triplicate for each strain. The biofilms produced by the *hwp1*Δ/Δ strains had a tendency to partially or completely slide off the silicone square just prior to drying, and these squares were excluded from measurements. The data represent measurements of 3-5 biofilms.

### Statistical methods

Survival studies in BALB/c or C57BL/6 mice were analyzed for significance using the Log-rank (Mantel-Cox) test derived from Kaplan-Meier survival curves. Mean biofilm dry weights were analyzed by one-way ANOVA using Tukey’s multiple comparisons test assuming an alpha value of 0.05. Gut colonization levels (CFU/g of stool) were analyzed by one-way ANOVA of log-transformed data and applying Tukey’s multiple comparisons test. For all statistical analyses, a P value <0.05 was considered significant. Statistical analyses were performed using GraphPad Prism (v. 6.01, GraphPad Software, Inc).

## Results

### Disruption of *HWP1* in wild type *C. albicans*

An open question regarding the contribution of Hwp1 to virulence is whether the TG substrate reactivity of the protein has a role in disseminated candidiasis. To address this question, we generated new *hwp1* null strains in wild type *C. albicans* SC5314 to avoid unknown *in vivo* auxotrophic requirements that could complicate virulence studies [18,20]. Also, the use of SC5314 vs. another parental strain for gene knock-outs, BWP17 [36], obviated the manipulation of a strain generated post multiple rounds of transformations and with reported chromosome abnormalities [37–39]. Because chromosomal copy number affects virulence or fitness *in vivo* in confounding ways [40], *HWP1* was disrupted in SC5314 at both alleles using the *flp* recombinase system [17] (Figure S1). Two independent *hwp1*Δ/Δ strains were created (SCH1211 and SCH2822), and one disruptant, SCH1211, was corrected for *HWP1* expression at its native locus using the same *flp* recombinase molecular system (Figure 1, and Information S1 and S2). Re-introducing *HWP1* also restored germ tube surface expression of Hwp1 (Information S3) and the amount of protein paralleled the expression of a single allele (compare *HWP1* RNA in lanes 1 vs. 3 in Figure 1; compare SC5134 and HR615 fluorescence brightness in S3). 

### The role of Hwp1 in disseminated candidiasis

#### Virulence of the *hwp1*Δ/Δ strain in Tgm2^-/-^ mice

**Figure 2:**
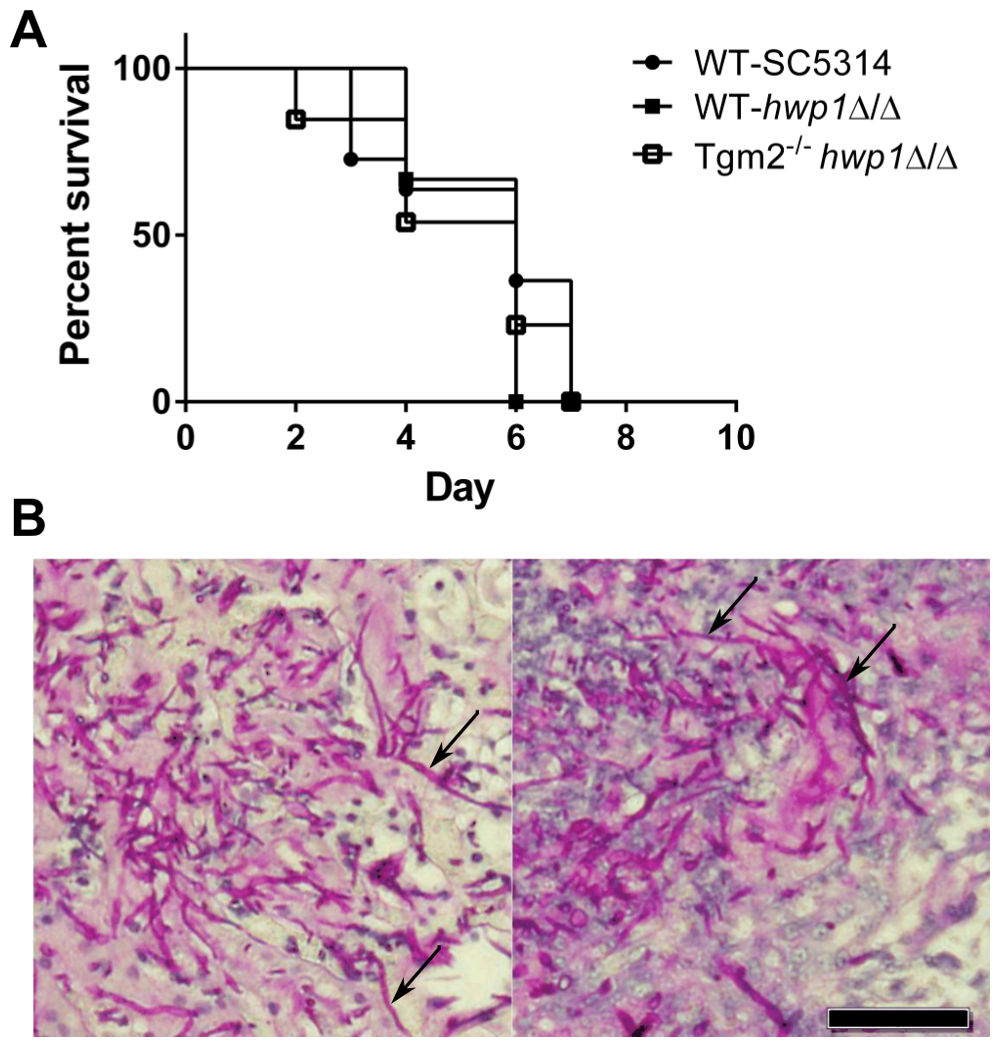
The *hwp1* SCH1211 null strain retains virulence in wild type and Tgm2^-/-^ mice. (**A**) Wild type C57BL/6 (n=6) and Tgm2 knock-out (n=13) mice were infected with 3-3.5 x 10^5^ SCH1211 yeasts and observed for signs of illness over an eight day period. C57BL/6 mice (n=11) infected with the wild type parental strain, SC5314, served as comparator. The survival kinetics among the groups of mice was indistinguishable (P>0.05). The median survival day for all groups was 6 days. (**B**) Histological analysis of kidneys from C57BL/6 (WT, left panel) or Tgm2 knock-out (Tgm2^-/-^, right panel) mice sacrificed 3-days post infection with SCH1211. Kidney sections were stained with period acid Schiff to visualize fungal elements. Arrows point to hyphae penetrating the tissue. Size bar=50 µm.

The newly constructed *hwp1*Δ/Δ strain SCH1211 and the parental SC5314 were tested for virulence [35] in wild type C57BL/6 and Tgm2^-/-^ mice to determine whether TG activity participated in *C. albicans* tissue infection such as in the kidneys where TG2 is active [16]. Disseminated candidiasis was established by intravenous injection of yeasts; 3.5 x 10^5^ yeasts per animal as per published methods [35]. Wild type or Tgm2^-/-^ mice infected with SCH1211 succumbed to candidiasis at equal rates and at the same rate as wild type C57BL/6 mice infected with SC5315 (Figure 2A). Median survival rates for all groups of mice were 6 days. Histological analysis of kidneys from wild type and Tgm2^-/-^ mice infected with SCH1211 revealed the invasion of tissue to similar degrees (Figure 2B). We observed identical survival kinetics when we tested a second, independently generated *hwp1*Δ/Δ strain SCH2283 in wild type and Tgm2^-/-^ mice (Supporting Information, Figure S4). The use of an independent *hwp1*Δ/Δ strain discounted the possibility of a SCH1211-specific phenotype independent of the *HWP1* deletion. The similar rate of survival among all the mice suggested that Hwp1 was dispensable for disseminated candidiasis in the C57BL/6 genetic background.

#### Virulence of the *hwp1*Δ/Δ strain in BALB/c mice

**Figure 3:**
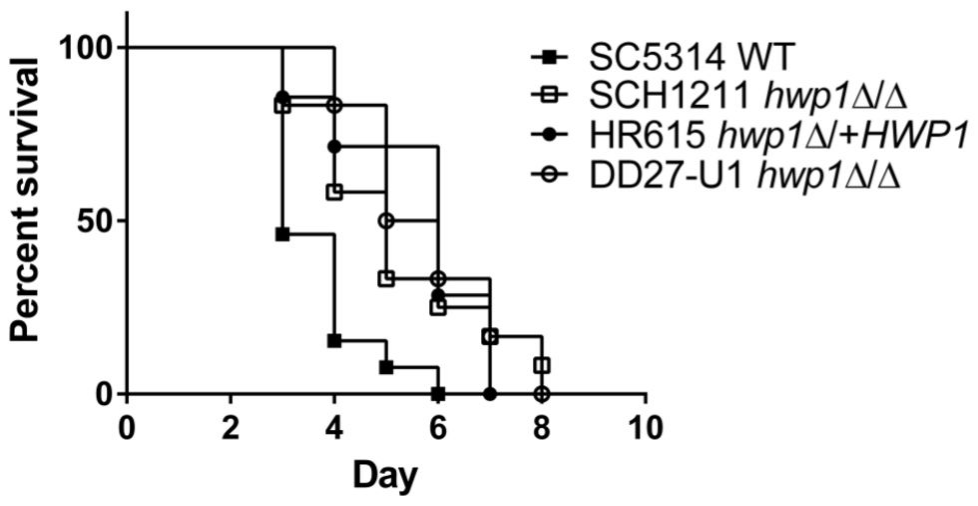
*hwp1* null strains display attenuated virulence in BALB/c mice. BALB/c mice infected with *hwp1*Δ/Δ strains SCH1211 (n=11) or DD27-U1 (n=6) were attenuated in virulence in intravenously infected BALB/c mice. Reintroducing *HWP1* at its native site in SCH1211 (HR615, n=7) did not restore full virulence of the strain to wild type levels (SC5314, n=13). The rate of survival among the strains deleted for *HWP1* (SCH1211 and DD27-U1) or expressing a single allele (HR615) was statistically different (P<0.05) relative to the wild type SC5314.

As a consequence of the virulence results using C57BL/6 mice, we wanted to re-test the designation of Hwp1 as a virulence factor in disseminated candidiasis. Previous *HWP1* deletion virulence studies were performed in BALB/c mice utilizing strains generated in CAI4 [27] with *URA3* at the *ENO* locus [15] or at its native locus [29]. We repeated the virulence studies in BALB/c mice with the newly created strains, and found that the absence of *HWP1* expression attenuated the virulence of *C. albicans* (Figure 3) that was statistically different from that of SC5314 (Log-rank test, P=0.01). The median survival of mice infected with SCH1211 was 5 days vs. 3 days for mice infected with SC5314. Mice that received the reconstituted strain HR615 had a 6 day survival median and the rate of death was not different from that of mice infected with SCH1211 (P=0.87). We also tested the *hwp1*Δ/Δ strain DD27-U1 created in a CAI4 background with *URA3* at its native locus and corrected for the partial *IRO1* deletion [29]. Mice infected with DD27-U1 had a median survival of 5.5 days and a similar rate of death relative to SCH1211 (P=0.73) and HR615 (P=0.73). Our results here contrasted with our previous survival data that show a median survival of >10 days in BALB/c mice infected with *hwp1*Δ/Δ strains even though the inoculum was higher [15]. Our current results are in line with data reported by Skarkey, et al. [29] who show an attenuated virulence for DD27-U1 in BALB/c mice. Although survival rates of mice infected with *hwp1*Δ/Δ strains were statistically different from that of SC5314, the relatively small boost in the survival rate of mice infected with the former strains appeared to diminish the biological significance of Hwp1’s role in disseminated candidiasis. 

### Hwp1 and *in vitro/in vivo* biofilms

#### Formation of biofilms on a silicone surface

**Figure 4:**
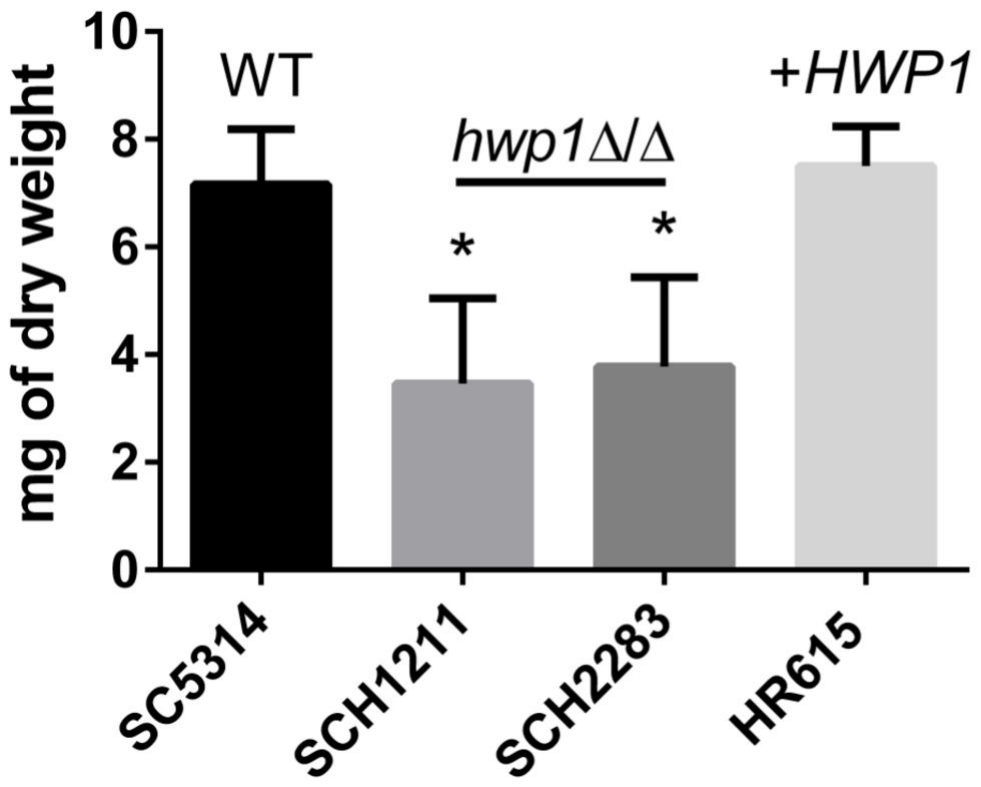
Biofilm formation is defective in newly created *hwp1* null strains. *C. albicans* strains were induced to form biofilms on the surface of silicone squares for 60 hr in Spider medium at 37°C. Two independently created *hwp1* null strains, SCH1211 and SCH2283, generated less biomass that were less adherent to the silicone squares. Reintroduction of a single *HWP1* allele into SCH1211 restored wild type biofilm biomass levels (HR615). The dry weights of the *hwp1* null mutants were statistically different relative to that produced by wild type SC5314 (asterisks, P<0.05; One-way ANOVA, multiple comparisons). The mean dry weight of HR615 was not statistically different relative to that produced by SC5314. The data represent means of 3-5 measurements from two independent experiments performed in triplicate.

In light of the virulence studies results, we tested other phenotypes associated with Hwp1. Hwp1 may have a significant role in the establishment of *C. albicans* at specific anatomical sites, e.g. the oral cavity, but have a minor role in invasive disease. Re-validating other Hwp1-associated phenotypes using our newly created *hwp1*Δ/Δ strains would bolster this hypothesis. A well-established function for Hwp1 is the mediation of *C. albicans* self-association in a three-dimensional colonial structure typical of biofims [3,21,22,41–43]. We used the silicone-square method [22,42] to quantify biofilm formation among the strains. Both *hwp1* null strains, SCH1211 and SCH2283, were defective in biofilm formation, and this defect was corrected by reintroducing a single copy of *HWP1* into SCH1211 (Figure 4). Our strains retained the *in vitro* biofilm defect phenotype observed by others [21], confirming the important role of Hwp1 in biofilm formation.

#### Formation of oral biofilms

Pseudomembranous oropharyngeal candidiasis lesions are considered a form of *in vivo* biofilm [3], and several animal models mimic this localized infection of the oral cavity [23,44–46]. These models are based on the superficial infection of murine tongues post oral inoculation of *C. albicans* in immunosuppressed or immunocompromised mice. The need for Hwp1 to form *in vivo* oral biofilms further supports the role of Hwp1 as a niche-specific virulence attribute required for colonization and local invasion of tissue. To test the capability of the *hwp1*Δ/Δ strain to form *in vivo* biofilms, BALB/c mice were orally inoculated [23] with SCH1211 and HR615, and sacrificed 3 days later to determine fungal burdens of the tongues. Two of 6 mice inoculated with SCH1211 were lost post-procedure; however the remaining 4 mice did not develop oral candidiasis (no CFU). In contrast, 6 of 7 mice infected with HR615 had infected tongues (mean CFU/g tongue, 4.2 x 10^5^ +/- 2 x 10^5^) at comparable levels reported for SC5314 [23]. Previous OPC studies in mice found that lack of *HWP1* expression impaired the strain’s ability to establish an infection of the tongue [3], and our results confirmed the *in vivo* biofilm defective phenotype of our *hwp1*Δ/Δ strain. Published data [13] together with our results here also demonstrated that *URA3* function did not influence the establishment and local invasion of oral tissue in mice further supporting the independent and key role of Hwp1 in OPC. Indeed, CAI4 (*ura3*Δ/Δ) is capable of producing OPC in immunocompromised gnotobiotic mice [47] showing that *URA3* function is nonessential in this infection model.

### Gut translocation studies

#### Intestinal colonization of C57BL/6 mice with *C. albicans* prior to translocation studies

**Figure 5:**
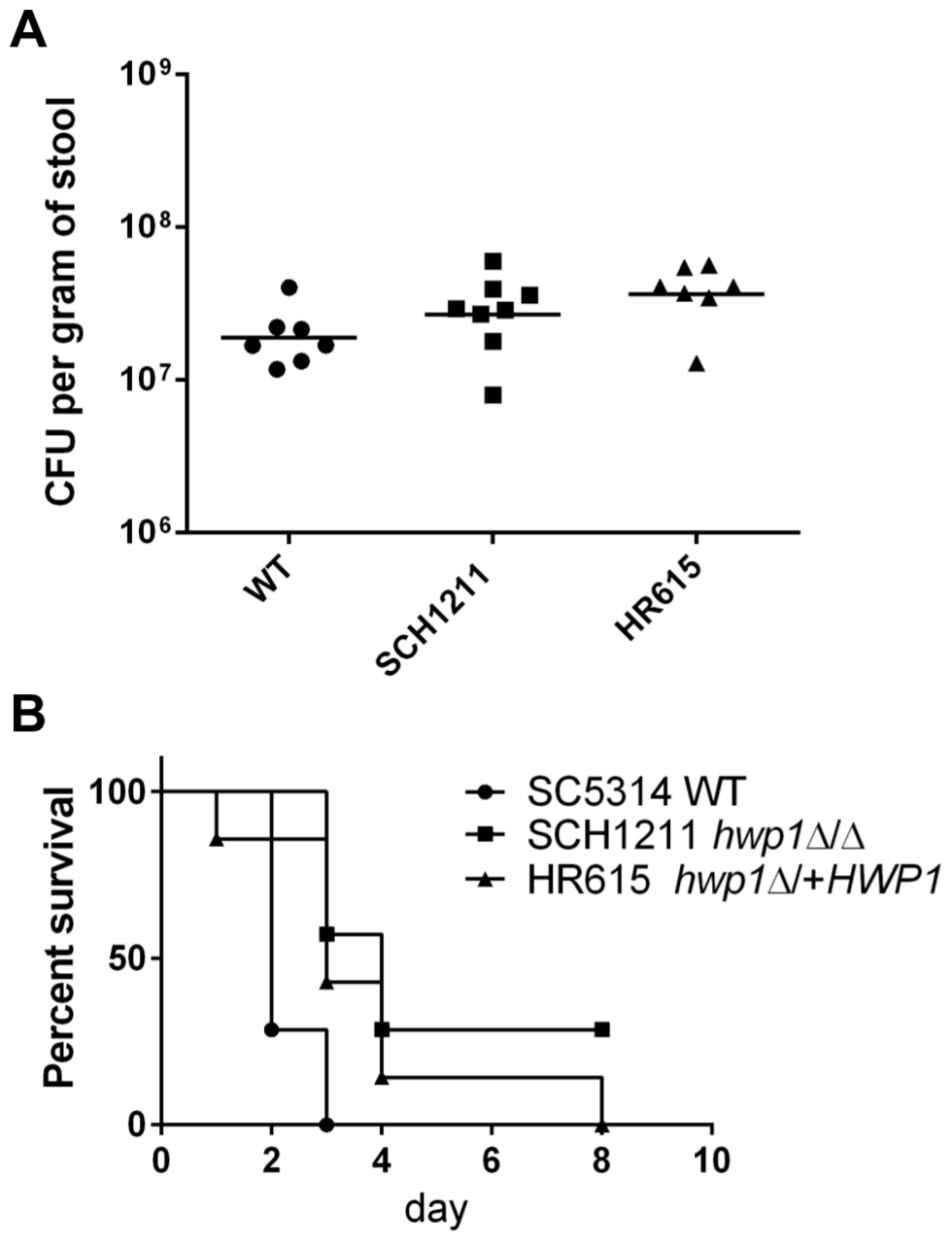
Hwp1 is required for full virulence in an animal model of gut translocation candidiasis. C57BL/6 mice were fed *C. albicans* in their drinking water to establish GI colonization followed by immunosuppression to induce neutropenia and intestinal mucosa damage. (**A**) Gut colonization levels are independent upon *HWP1* expression. Colonization levels in mice were assessed by measuring the fungal burden in the stools (CFU/g) of individual mice. Mean CFU/g of stool of mice fed SCH1211 (n=8) or HR615 (n=7) were not statistically different relative to SC5314 (WT, n=7). (**B**) Survival of mice post immunosuppression with cyclophosphamide. Mice colonized with the *hwp1* null strain SCH1211 were less virulent relative to SC5314 (P=0.003). Single expression of *HWP1* (HR615) did not restore wild type survival kinetics in mice (P=0.035), although none of the mice colonized with HR615 survived to the end of the observation period. *C. albicans* was recovered from the livers of all the mice at the time of sacrifice (data not shown), indicating translocation from the GI tract.

A common route of infection in hospitalized patients is the translocation of endogenous *C. albicans* from the intestinal tract into the blood stream associated with antibiotic use and hematological immunosuppression or chemotherapy that result in the loss of intestinal barrier function [48]. The requirement for Hwp1 to promote dissemination of *C. albicans* from the GI tract is unknown, and may define a new role for the fungal adhesin. We used the murine model that mimics this route of infection developed by Koh and colleagues [24]. Mice are treated with antibiotics and fluconazole to allow the colonization of *C. albicans* in their GI tracts prior to immunosuppression. We found that all three *C. albicans* strains, SC5314, SCH1211 and HR615, colonized the intestinal track of C57BL/6 mice to similar levels (Figure 5A). The median SC5314 CFU/g of stool, 2.03 x 10^7^, was comparable to that achieved in C3H/HeN mice (2.24 x 10^7^ CFU/g of stool) [24]. SCH1211 and HR615 colonized the mice at slightly higher numbers (3.06 x 10^7^ and 3.94 x 10^7^ CFU/g of stool, respectively; the levels of gut colonization were not statistically different among the groups by one-way ANOVA, Tukey’s multiple comparisons test, alpha=0.5) suggesting that Hwp1 was not influential in gut colonization of mice. Hwp1 is also not a factor in colonizing the GI tract of gnotobiotic mice [13]. 

#### Gut translocation and dissemination of *C. albicans* to the liver

The gut-colonized mice were treated with intraperitoneal injections of cyclophosphamide to induce immunosuppression/mucosal damage, and allow translocation of *C. albicans* into the blood stream and dissemination to the liver. We modified the method of Koh et al. [24] to add an additional cyclophosphamide injection after pilot experiments revealed inconsistent translocation of *C. albicans* to the liver of mice treated with the published number of cyclophosphamide injections. All of the mice colonized with strains expressing at least one allele of *HWP1* (n=14) died by day 8 post-immunosuppression while 2 of the 7 mice colonized with the *hwp1*Δ/Δ strain survived to the end of the study (Figure 5B). The survival rate between SC5314 and SCH1211-colonized mice was statistically different (P=0.003); however the survival rates of mice harboring SCH1211 or HR615 were not different from each other (P=0.21). *C. albicans* was recovered from the livers of all the mice at the time of death demonstrating translocation from the GI tract to the organ independent of *HWP1* expression. The mice colonized with SCH1211 did not have statistically different median CFU/g of liver compared to mice colonized with SC5314 or HR615 (one-way ANOVA with Tukey’s multiple comparisons test, alpha=0.5; data not shown). These results suggested that wild type hyphal surface levels of Hwp1 were needed for rapid translocation of *C. albicans* from the mouse gut into the blood stream. However, once translocation had occurred, the lack of Hwp1 did not prevent dissemination to and establishment of *C. albicans* in the liver.

## Discussion

 The studies here were initiated to address the role of TG-mediated reactions as a possible mechanism for the requirement of Hwp1 in disseminated candidiasis. We expected to protect mice infected with the *hwp1*Δ/Δ strain from death in a Tgm2^-/-^ background relative to wild type mice. However, our results discounted a TG-mediated mechanism in the virulence of *C. albicans* driven by Hwp1. The equal rates of deaths among the wild type C57BL/6 and Tgm2^-/-^ mice, regardless of the infecting strain, supported the null hypothesis. Further, histological analysis of infected kidneys revealed invasion of tissue by the *hwp1*Δ/Δ strain independent upon Tgm2 expression status. However, the possibility existed that transamidation-unrelated factors confounded the survival data. Tgm2^-/-^ mice are less sensitive to bacterial endotoxin [49], and TG2 is elevated during inflammatory processes [50–53]. A dysregulated immune response may have obscured any benefit in survival in Tgm2^-/-^ mice. The similar rates of death observed in C57BL/6 mice infected with either SC5314 or SCH1211 lessened the association between Hwp1 and virulence (whether the protein participated in transamidation reactions in tissue or not). And although the use of Tgm2^-/-^ mice had limitations, the lack of TG2 activity was not sufficient to improve survival to a significant level even in the absence of Hwp1. Thus, we concluded that host tissue TG2 activity in organs such as the liver and kidneys [16] was dispensable or played a small role in the successful dissemination and tissue invasion by *C. albicans*. Host transamidation activity did not appear to promote dissemination of *C. albicans* via reactions involving Hwp1.

 The wild type virulence of the *hwp1*Δ/Δ strains in C57BL/6 mice was an unexpected result of our studies. C57BL/6 and BALB/c are considered to be less susceptible to intravenous *C. albicans* challenge among mice of different genetic backgrounds tested in this model of candidiasis [54–58], and we observed similar survival kinetics between the mouse strains injected with the wild type SC5314. However, BALB/c and C57BL/6 mice can produce different immunological responses to *C. albicans* infections and complicate the interpretation of virulence data [59–63]. The susceptibility to *C. albicans* infection can be difficult to qualify when survival kinetics reveal an attenuated virulence in a given mouse strain. The inability to observe the same attenuated virulence of SCH1211 in C57BL/6 mice may reflect differences of genetic effects between C57BL/6 and BALB/c mice that govern susceptibility to candidiasis. At least two and perhaps more genetic loci are linked to susceptibility to infection with *C. albicans* [58], and how these factors affect survival kinetics is unknown. This can be a caveat when working with C57BL/6 transgenic knock-out mice which are not historically used in *C. albicans* virulence studies. If the role of Hwp1 in disseminated candidiasis was weak at best, this effect was masked in the C57BL/6 genetic background. 

Consideration of the virulence data independently of mouse strain suggested that Hwp1 was not a major factor in dissemination of *C. albicans* regardless of Tgm2 expression. The attenuated virulence of the *hwp1* null strain observed in BALB/c mice, not corrected by re-introducing a single allele of *HWP1*, has been noted by others [29], and further supports a diminished role for Hwp1 in disseminated candidiasis. Previous virulence studies utilizing *hwp1*Δ/Δ strains [1,15] attributed a greater effect to Hwp1 in systemic candidiasis due to the large shift in survival rates and to the survival of a subset of mice to the end of the study. These latter *hwp1*Δ/Δ strains were created in CAI4 and not corrected for the large deletion that encompasses *URA3*, a region of *IRO1*, a putative transcriptional factor, and perhaps the 3’ untranslated region of ORF19.1717 ([20] and www.candidagenome.org). Loss of a functional *IRO1* is known to affect the virulence of *C. albicans* [64]. Full restoration of ORF19.1717-*URA3*-*IRO1* in CAI4 generates an *hwp1*Δ/Δ strain (DD27-U1) with attenuated virulence BALB/c mice [29], a phenotype that we recapitulated here and observed with our newly created *hwp1*Δ/Δ strains in SC5314. Together, the data suggested that strains not fully restored at the *URA3* locus confused previous results of virulence studies using *hwp1*Δ/Δ strains and attributed a greater virulence function to Hwp1. *URA3*/CAI4-related effects on virulence of *C. albicans* has been addressed in the literature [15,18,20,29,65,66]; however the studies here highlighted once again the importance of selecting the appropriate tools to address virulence questions. Small or nuanced contributions to phenotypes can be missed *in vivo* by confounding host and fungal factors. The data also highlighted the complex relationship between *C. albicans* and the mammalian host, likely defined by the host’s niche and genetic factors that modulate infection. 

*HWP1* is part of a core of 8 genes induced during filamentation of *C. albicans* that is independent of media [67]. *HWP1* together with 3 other filamentation core genes, *ALS3*, *ECE1*, and *RBT1*, code for hyphal wall proteins that function in biofilm formation [21,22,68]. Expression of *ALS3*, *ECE1*, *HWP1* and *RBT1* is induced in fungal cells within biofilms, when they are in contact with plastic and with oral epithelial cells [67]. *ALS3* and *RBT1* deletion mutants have no role or a reduced effect, respectively, on the virulence of *C. albicans* in BALB/c mice in a systemic model of candidiasis [69,70]; presently there are no reports describing an *ECE1* murine virulence study. Hwp1, Rbt1, Als3p are required for *C. albicans* mating at wild type levels [21,42], another type of self-interaction in addition that which occurs in “pathogenic” biofilms [71]. The phenotypic parallels between *hwp1*Δ/Δ *als3*Δ/Δ and *rbt1*Δ/Δ strains suggest that these gene products function primarily for self (Hwp1, Als3p and Rbt1p) and in host-fungal interactions on the surface of epithelial or endothelial cells (Hwp1 and/or Als3p, respectively) at host niches (oral and esophageal mucosa) where a biofilm phenotype is the dominant “infectious” morphology. However, the role of these proteins to individually promote candidiasis that involves deep tissue invasion in mice appears to be minimal. Finally, *C. albicans* clinical isolates expressing an allele of *HWP1* (*HWP1-2*) form poor biofilms *in vitro* but are nevertheless recovered from the blood stream of patients [72], supporting our conclusions here that Hwp1 is important for biofilms but is not important in promoting systemic candidiasis. 

Wild type levels of Hwp1 were needed for the rapid translocation of *C. albicans* from the gut into the blood stream and infection of murine livers. However, the *hwp1* null strain was able to colonize murine guts at wild type levels similarly to that observed for *rbt1* deletion mutants [73]. Both of these genes are expressed in *C. albicans* cells found in the cecum of C57BL/6 mice [73,74] although the cells are predominantly in the yeast morphology. *HWP1* expression has also been detected in fungal cells recovered from the oral cavities of asymptomatic individuals [75] presumably growing as a commensal organism (yeasts) of the mouth. It is not clear whether *HWP1* expression originates in yeast cells at these anatomical niches or in low numbers of hyphal cells that are part of the commensal population. *C. albicans* cells unable to form filaments and express *HWP1* (*cph1*Δ/Δ *efg1*Δ/Δ) [76,77] do not translocate from the mouse gut to the liver as well as SC514; in contrast, cells locked in the filamentous morphology (*tup1*Δ/Δ) appear more virulent relative to wild type cells in this animal model of candidiasis [24]. These results did not distinguish between the ability of *C. albicans* to undergo morphological transitions or the expression of adhesins (e.g. Hwp1, Als3p) associated with the hyphal morphology as factors affecting gut translocation. Because *hwp1* null strains are not deficient in filamentation ([1] Figure 2 and Figure S3), we were able to consider hyphae formation and *HWP1* expression as separate variables in our studies. The results here implied that expression of adhesins and perhaps other proteins associated with the hyphal form are the key variables essential for *C. albicans* translocation. The mechanism by which Hwp1 aids translocation is not yet understood but we propose two models that are not mutually exclusive: (A) a critical amount of *C. albicans* self-aggregation is necessary to achieve a threshold fungal burden traversing the damaged intestinal mucosa from the lumen into the blood stream. Dissemination to the liver of the *hwp1* null strain was delayed as a consequence of decreased cell numbers trafficking from the GI tract to the blood stream. In support of this hypothesis, the flocculent *tup1*Δ/Δ cells are more virulent in this gut translocation candidiasis model relative to wild type even when gut colonization levels are two logs below wild type [24]. (B) Alternatively, Hwp1 contributed to interactions with epithelial cells lining the intestinal mucosa and the lack of Hwp1 hampered the initial binding and subsequent translocation of the fungus into the blood stream. Hwp1also participates in adhesion of *C. albicans* to oral epithelial cells in a non-TG dependent manner [1], therefore it is plausible that the lack of such fungal-host interactions may affect the kinetics of GI translocation. 

Expression of a single allele of *HWP1* in HR615 restored biofilm formation both *in vitro* and *in vivo* but did not fully correct the *hwp1* null phenotype in two out of three animal models that involved dissemination via the blood stream. The factors that control wild type dissemination of *C. albicans* are likely different from those that influence the establishment of superficial biofilms on mucosal surfaces. The results observed with HR615 suggested that Hwp1 may participate in yet undefined functions that aid dissemination via the blood stream that require wild type Hwp1 expression levels. 

Taken together, our results here support a new functional model for Hwp1 and similar fungal adhesins: Hwp1 engenders *C. albicans* with niche-specific properties to form biofilms at oral/mucosal surfaces with localized penetration of the underlying tissue. Hwp1 and other hyphal proteins organize and maintain the three-dimensional structure of the biofilm which is held in place by cross-linking Hwp1 to the surface of oral epithelial cells by the action of host TG. Thus Hwp1 performs the dual function of self-aggregating *C. albicans* into pathogenic biofilms and adhesion of the biofilm/hyphal cells to the surface of host tissue via TG activity. *HWP1* gene expression differs from other niche-specific genes in that *HWP1* is normally expressed in hyphal cells regardless of host niche. Many of the currently known differentially-expressed niche-specific genes code for enzymes (e.g. *SAP*s, phospholipases, and carbon metabolic proteins) [78–87] that enhance the growth fitness of *C. albicans* as infection takes hold and progresses at given anatomical sites. Hwp1 plays an important niche-defined structural role in *C. albicans in vivo* colonial morphology that is not specifically associated with fungal fitness or invasive growth advantage. Ultimately, the ability to combine fitness-enhancing niche-specific gene expression with the appropriate infectious colonial morphology adapts *C. albicans* for survival and growth at ecologically diverse anatomical sites found within its mammalian host. 

## Supporting Information

Table S1 **DNA primers used for generating the *HWP1* disruption and reconstitution cassettes.** Oligonucleotides used to generate the *hwp1*Δ:*SAT1* and *HWP1*:*SAT1* gene cassettes for the construction of deletion and reconstitution (put-back) strains in pSFS1, respectively. Underlined nucleotides introduce an ApaI site at the 5’ end of the amplicon. Double underlined nucleotides introduce XhoI sites; nucleotides in bold introduce a SacII site. The nucleotides in small letters indicate the SacI site in the 3’ region downstream of *HWP1*.
(DOCX)

Figure S1 **Construction of the *hwp1* deletion and reconstituted strains.** (**A**) Schematic of the *HWP1* genomic locus (middle construct) with integration of the *HWP1*-disruption cassette. NdeI restriction sites are shown in bold above the DNA constructs. The PCR amplicon used to detect *HWP1* sequences in Southern blots is labeled “*HWP1* probe”. The size marker at top indicates 300 nt. Gene designations: *CaFLP*, sequences coding for the *C. albicans* flp recombinase; *CaSAT1*, sequences coding for *C. albicans* nourseothricin resistance. The arrow above the inducible *SAP2* promoter (*SAP2p*) indicates the direction of transcription of the *CaFLP* gene. Dashed green lines indicate the double crossover event leading to integration of the disruption cassette (bottom schematic). (**B**) Reconstitution of *HWP1* expression at its native locus. *hwp1*Δ::*FRT* at the *HWP1* genomic locus (bottom) with integration of the reconstitution cassette (top). Dashed green lines indicate the sites of the double crossovers restoring expression of *HWP1*. Size marker, 300 nt. The schematics were generated using Gene Construction Kit (v. 3.5, Texco BioSoftware, Inc.).
(TIF)

Figure S2 **Southern blot analysis of the *HWP1* deletion and reconstitution strains used in this study.** SC5314 and subsequent *HWP1* deletion/reconstitution derivatives are shown in order from left to right. The expected *HWP1*-hybridizing NdeI DNA fragments (arrows at left) are as follows: *HWP1*, 6.3 Kb, *hwp1*Δ::*SAT1*, 3.9 Kb, *hwp1*Δ::*FRT*, 4.5 Kb, *HWP1*::*SAT1*, 6.3 Kb, and *HWP1*::*FRT*, 6.7 Kb . The strains in bold lettering were used in the studies described here.
(TIF)

Figure S3 **Expression of Hwp1 on the surface of *C. albicans* germ tubes.** The primary amine, 5-(biotinamido)pentylamine and human recombinant TG2 were used to visualize Hwp1 on the fungal surfaces (see Materials and Methods). The expression level of Hwp1 on the surface of HR615 appeared decreased relative to SC5314 consistent with the introduction of a single copy of *HWP1* at its native locus in SCH1211 (see Figure 1, northern analysis of SCH1211 and HR615). Left column, light images; right column, FITC and DAPI (nuclear staining) images combined. Size bar, 20 µm.
(TIF)

Figure S4 **Virulence of SCH2283 (*hwp1*Δ/Δ) in C57BL/6 wild type and Tgm2 knock-out mice.** Survival curves of wild type (n=5) or Tgm2^-/-^ (n=7) mice injected with a second *HWP1* deletion strain, SCH2283. Survival rates between the animal groups were indistinguishable, P=0.86. The median survival for both groups of mice was 3 days.
(TIF)
