# Supplementary material for: Niche-Specific Requirement for Hyphal Wall protein 1 in Virulence of Candida albicans
Source: PLoS One. 2013 Nov 8;8(11):e80842. doi: 10.1371/journal.pone.0080842 (PMC3832661; doi:10.1371/journal.pone.0080842)
Supplement: Table S1 — DNA primers used for generating the HWP1 disruption and reconstitution cassettes. Oligonucleotides used to generate the hwp1Δ:SAT1 and HWP1:SAT1 gene cassettes for the construction of deletion and reconstitution (put-back) strains in pSFS1, respectively. Underlined nucleotides introduce an ApaI site at the 5’ end of the amplicon. Double underlined nucleotides introduce XhoI sites; nucleotides in bold introduce a SacII site. The nucleotides in small letters indicate the SacI site in the 3’ region downstream of HWP1. (DOCX) [file pone.0080842.s001.docx]

| Primer name | Sequence |
| --- | --- |
| H1Ap | 5’-TCGGGCCCCAAGGAATTCGGAAATTCTGACG-3’ |
| H2Xh | 5’-CGGCTCGAGGCGATAGCAATAAGTTGAGCAGTTG-3’ |
| H3SII | 5’-TCC**CCGCGG**GACTTTCGGTGCTGCTATTATTGG-3’ |
| H4 | 5’-GAAGATACCATAAGCTACAgagctcAC-3’ |
| H5Xh | 5’-CCGCTCGAGAAGGATTATAGAATCAAACTTGGGG-3’ |
