# Supplementary figures and images for: Niche-Specific Requirement for Hyphal Wall protein 1 in Virulence of Candida albicans
Source: PLoS One. 2013 Nov 8;8(11):e80842. doi: 10.1371/journal.pone.0080842 (PMC3832661; doi:10.1371/journal.pone.0080842)

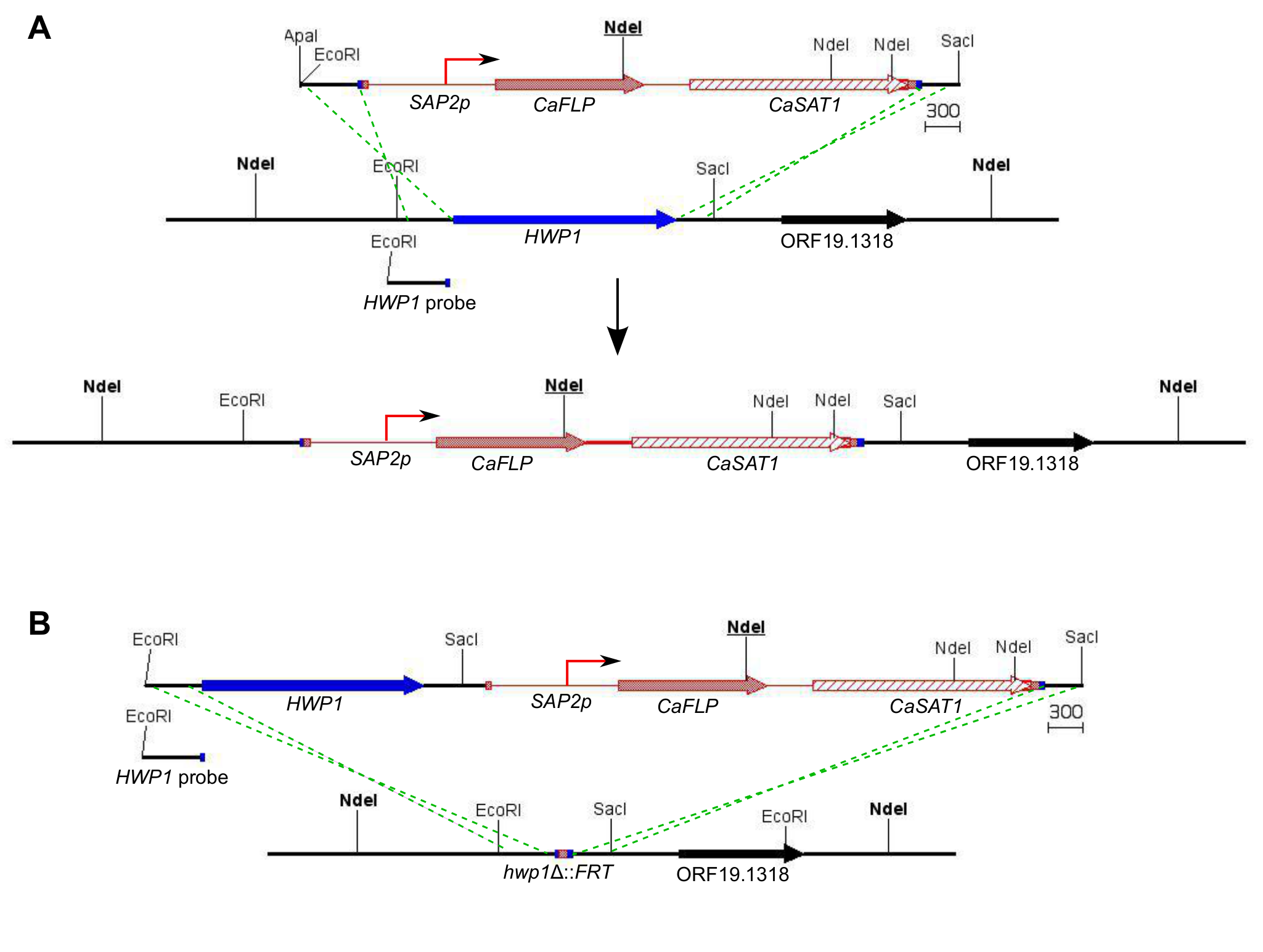

Supplement: Figure S1 — Construction of the hwp1 deletion and reconstituted strains. (A) Schematic of the HWP1 genomic locus (middle construct) with integration of the HWP1-disruption cassette. NdeI restriction sites are shown in bold above the DNA constructs. The PCR amplicon used to detect HWP1 sequences in Southern blots is labeled “HWP1 probe”. The size marker at top indicates 300 nt. Gene designations: CaFLP, sequences coding for the C. albicans flp recombinase; CaSAT1, sequences coding for C. albicans nourseothricin resistance. The arrow above the inducible SAP2 promoter (SAP2p) indicates the direction of transcription of the CaFLP gene. Dashed green lines indicate the double crossover event leading to integration of the disruption cassette (bottom schematic). (B) Reconstitution of HWP1 expression at its native locus. hwp1Δ::FRT at the HWP1 genomic locus (bottom) with integration of the reconstitution cassette (top). Dashed green lines indicate the sites of the double crossovers restoring expression of HWP1. Size marker, 300 nt. The schematics were generated using Gene Construction Kit (v. 3.5, Texco BioSoftware, Inc.). (TIF) [file pone.0080842.s002.tif]

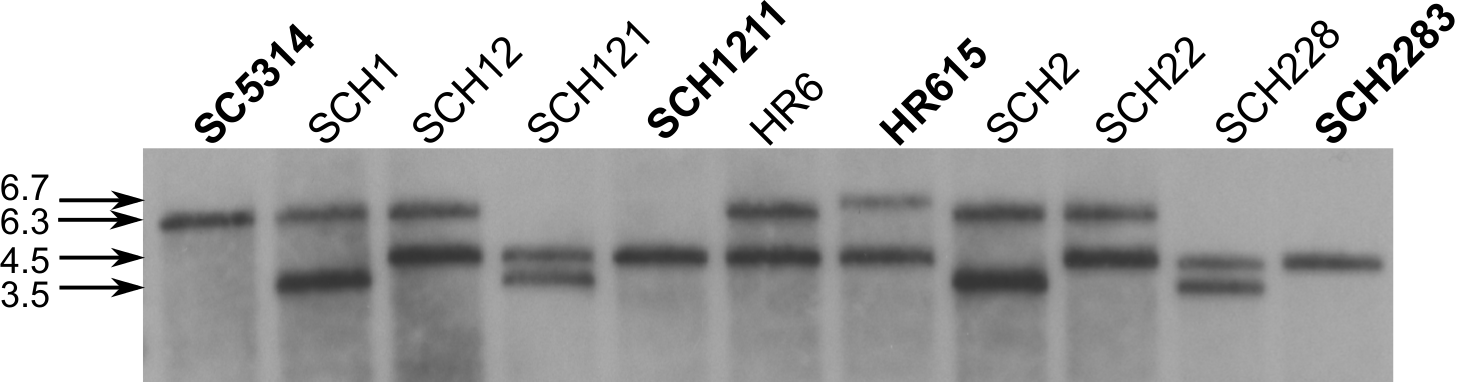

Supplement: Figure S2 — Southern blot analysis of the HWP1 deletion and reconstitution strains used in this study. SC5314 and subsequent HWP1 deletion/reconstitution derivatives are shown in order from left to right. The expected HWP1-hybridizing NdeI DNA fragments (arrows at left) are as follows: HWP1, 6.3 Kb, hwp1Δ::SAT1, 3.9 Kb, hwp1Δ::FRT, 4.5 Kb, HWP1::SAT1, 6.3 Kb, and HWP1::FRT, 6.7 Kb . The strains in bold lettering were used in the studies described here. (TIF) [file pone.0080842.s003.tif]

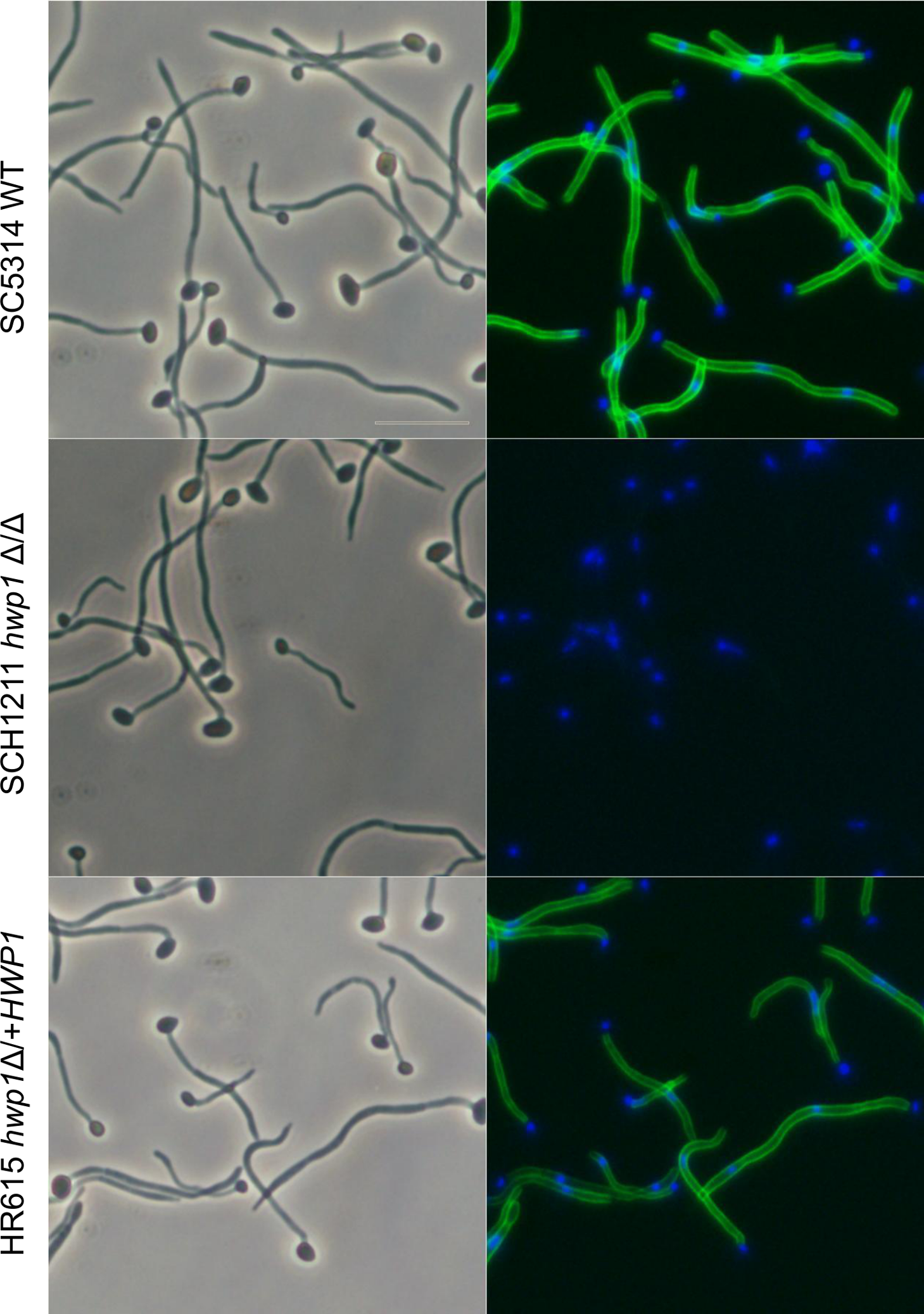

Supplement: Figure S3 — Expression of Hwp1 on the surface of C. albicans germ tubes. The primary amine, 5-(biotinamido)pentylamine and human recombinant TG2 were used to visualize Hwp1 on the fungal surfaces (see Materials and Methods). The expression level of Hwp1 on the surface of HR615 appeared decreased relative to SC5314 consistent with the introduction of a single copy of HWP1 at its native locus in SCH1211 (see Figure 1, northern analysis of SCH1211 and HR615). Left column, light images; right column, FITC and DAPI (nuclear staining) images combined. Size bar, 20 µm. (TIF) [file pone.0080842.s004.tif]

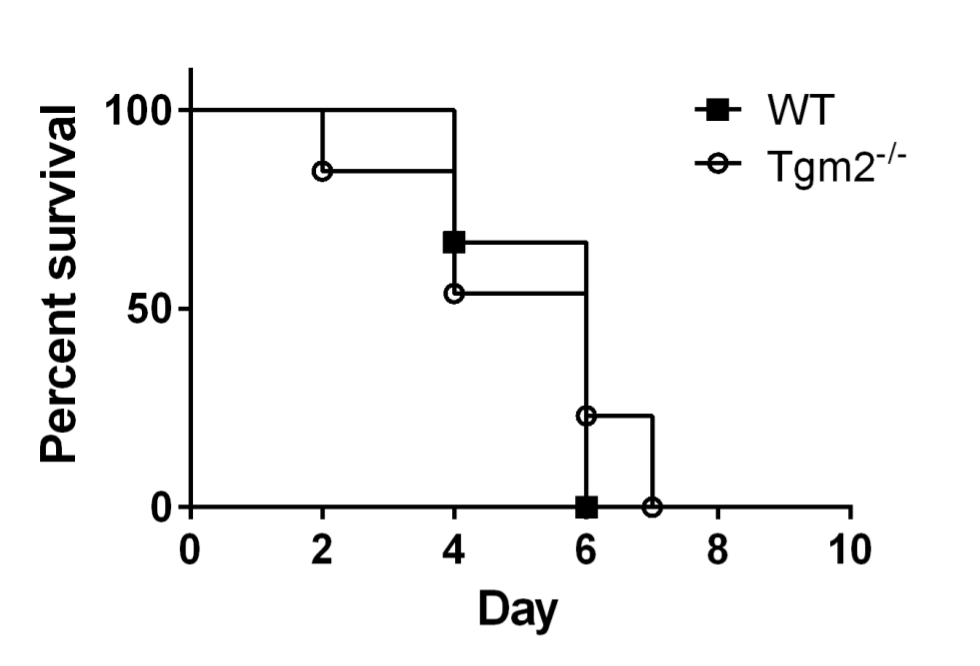

Supplement: Figure S4 — Virulence of SCH2283 (hwp1Δ/Δ) in C57BL/6 wild type and Tgm2 knock-out mice. Survival curves of wild type (n=5) or Tgm2-/- (n=7) mice injected with a second HWP1 deletion strain, SCH2283. Survival rates between the animal groups were indistinguishable, P=0.86. The median survival for both groups of mice was 3 days. (TIF) [file pone.0080842.s005.tif]
